# Nutrient Content and Sensory Acceptability of Home‐Based Therapeutic Food to Treat Children 6–59 Months With Moderate Acute Malnutrition

**DOI:** 10.1002/fsn3.70223

**Published:** 2025-05-07

**Authors:** Gashaw Abebaw, Welday Hailu, Tefera Belachew

**Affiliations:** ^1^ School of Nutrition, Food Science and Technology Hawassa University Hawassa Ethiopia; ^2^ Food Process Engineering Department College of Engineering and Technology, Wolkite University Wolkite Ethiopia; ^3^ Nutrition and Dietetics Department Faculty of Public Health, Jimma University Jimma Ethiopia

**Keywords:** moderate acute malnutrition, nutritional properties, therapeutic food

## Abstract

Moderate acute malnutrition (MAM) among children under 5 years old has been a daunting problem of public health significance in Ethiopia over the past half century, with its magnitude increasing over time. With dwindling global resources, the preparation of local solutions that can help to curb this problem is critically important. The objective of this study was to develop and analyze eight home‐based therapeutic foods to treat MAM in children aged 6–59 months. One‐way analysis of variance (ANOVA) was used to analyze differences in means with ± standard deviation of nutrient measurements among the samples. The nutrient contents ranged from 4.56% to 8.79% for moisture, 28.06% to 34.62% for fat, 10.03% to 13.91% for protein, and for energy, 498.31 kcal to 529.81 kcal/100 g of edible portion. The mineral contents ranged from 100.47 mg to 115.51 mg for calcium, 5.01 mg to 6.74 mg for zinc, 8.39 mg to 11.34 mg for iron, 544.15 mg to 661.54 mg for potassium, and 442.54 mg to 451.84 mg for phosphorus contents is adequate. The peanut (P), chickpea (C), maize (M), and orange flesh sweet potato (OFSP) (PCMOFSP^4^) with the highest portion of peanut seed flour had significantly the highest amounts of protein, fat, calories, iron, zinc, and potassium. These results were within the recommended range of required nutrients for the treatment of children with MAM. Therefore, home‐based therapeutic food may be used for the management of children with MAM. Enrich the formulation with micronutrients such as ascorbic acid (vitamin C) and calcium. Policies should prioritize advancing the creation of locally sourced, home‐based therapeutic foods to address MAM in children, while also fostering stronger collaboration between agricultural and nutrition sectors to ensure that sustainable, accessible solutions are recommended.

## Graphical abstract

Develop and analyze eight home‐based therapeutic foods to treat moderate acute malnutrition (MAM) in children aged 6–59 months. Peanut‐enriched formulations showed the highest concentrations of protein, fat, calories, iron, zinc, and potassium compared to other blends. Locally prepared therapeutic foods show potential for treating MAM in children.

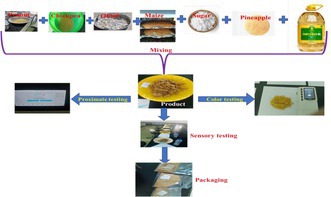

## 1 Introduction

Malnutrition is responsible for millions of deaths and disabilities globally each year (Jenfa et al. 2024), with undernutrition remaining a critical issue, particularly in developing countries (Cossa‐Moiane et al. 2024). Undernutrition is widely acknowledged as a severe global public health challenge (Chiopris et al. 2024). Annually, an estimated 18.7 million children experience severe acute malnutrition (SAM), while 51.5 million children under the age of five years are affected by moderate acute malnutrition (MAM) worldwide (Sigh et al. 2018). According to Arogundade et al. (2023), a child succumbs to malnutrition and infection every 15 s. UNICEF data further indicate that, in Least Developed Countries, 38% of children aged 1 to 59 months experience moderate to severe stunting, 10% suffer from wasting, and 23% are underweight. While undernutrition has shown a gradual decline in developed nations, it continues to rise in many developing countries worldwide (Black et al. 2013). Schoonees et al. (2019) describe undernutrition as a condition stemming from persistent infections combined with inadequate food intake, both quantitatively and qualitatively. Gupta et al. (2015) reported that undernutrition can be classified as acute or chronic, based on the duration of inadequate food intake and infection exposure.

The previously studied research done in the treatment of MAM now involves the use of some dietary supplements, such as lipid‐based nutrient supplements (AlOudat et al. 2021), BP5 biscuits (UNICEF 2015), and corn‐soy blend (CSB) (Nane et al. 2020). The most popular of these interventions is enriched blended flours, especially CSB when made into porridge (Medoua et al. 2016). Even if they are common, there are still issues, including high commodity prices and doubts regarding their long‐term viability and sustainability (Nane et al. 2020). According to Makori et al. (2024), appropriate feeding of nutrient‐dense, locally accessible foods has been demonstrated to be successful in the nutritional management of moderate acute malnutrition (MAM) at the home level. Foods that are readily available locally, accessible to everyone, and contain the required levels of nutrients can be used to create these dietary supplements (Handayani et al. 2022).

The challenges faced in MAM management include the high unit cost of products, having low coverage of programs, focusing on generalized prevalence rates rather than season‐specific incidence rates, and frequent high defaulting (Nane et al. 2022). This study was motivated by the limitations of current home‐based therapeutic food formulations, which predominantly target SAM or rely on ingredients that lack regional accessibility or cultural relevance in specific populations. To address these gaps in the development and evaluation of home‐based therapeutic foods specifically designed for MAM, particularly formulations that prioritize locally available, cost‐effective, and culturally acceptable ingredients. The development of nutritionally adequate and culturally acceptable therapeutic foods for managing MAM has been a focus of recent research, particularly formulations that utilize locally available ingredients (Lelijveld et al. 2020). Therefore, home‐based therapeutic food made from locally available, nutritionally dense foods might have a positive effect on treating MAM children (Choudhury et al. 2018). Locally available ingredients are affordable and can supply essential nutrients for children with MAM to recover effectively (Eloho et al. 2017). Local food materials can be cheap and provide essential nutrients required for the successful recovery of children with MAM. To the best of the literature, there has not been any local development of home‐based therapeutic foods from Ethiopia, specifically for MAM treatment. Meanwhile, the WHO advises utilizing locally available, nutrient‐dense foods for managing MAM in children. In this study, peanut, chickpea, maize, and orange flesh sweet potatoes were used to formulate locally produced therapeutic foods. The objective of this study was to develop and evaluate the physicochemical, nutritional profile, and sensory acceptability of home‐based therapeutic foods to treat children 6–59 months with MAM.

## 2 Materials and Methods

### 2.1 Selection of Food Ingredients

All selected food ingredients, such as peanut, chickpea, maize, orange‐fleshed sweet potato, sunflower, sugar, and pineapple powder were collected from local markets in Wolkite Town, Gurage Zone, Central Ethiopia. The ingredients were chosen based on local availability, affordability, seasonal accessibility, low cost, quality, and nutritional richness, particularly their high protein, energy, and vitamin A content. Peanuts and chickpeas provide plant‐based protein and healthy fats, while orange‐fleshed sweet potato (OFSP) supplies β‐carotene, a critical nutrient for immune function in malnourished children. Pineapple powder was included to enhance flavor (ensuring child acceptability) and vitamin C content, improving palatability for sensory acceptance. Sugar and sunflower improve texture, palatability, essential fatty acids, and energy.

### 2.2 Used Chemicals

The standards and reagents used included the Folin–Ciocalteu reagent (2, Sigma Aldrich, USA), catechin hydrate (≥ 96.0%, Sigma Aldrich, China), gallic acid (97.5%–102.5%, Sigma Aldrich, China), 2,2‐diphenyl‐1‐picrylhydrazyl (DPPH, Sigma Aldrich, Germany), quercetin (≥ 98%, Sigma Aldrich, Germany), vanillin (≥ 99.5%, UNI‐CHEM, Roth, France), phytic acid sodium salt hydrate (Sigma Aldrich, Switzerland), sodium carbonate (≥ 99.5%, Carl Roth GmbHH, Germany), aluminum chloride (99%, Loba Chemie, India), nitric acid (69%, Loba Chemie, India), and methanol (M.wt. = 34.02 g/mol, Biochem Chemopharma, France). Additional reagents included buffer solution (ready‐to‐use, Ethiopia), potassium hydroxide (90%, Turkey), sodium hydroxide pellets (Extra Pure, Alpha Chemika, India), methanol (99.9%, Taflen, Ethiopia), copper sulfate pentahydrate (CuSO_4_·5H_2_O, RANCHEM, Turkey), and soluble potato starch (Alpha Chemika, India). All the chemicals were used for analytical grade.

### 2.3 Material Collection and Preparation

The peanut, chickpea, orange‐flesh sweet potatoes, maize, sunflower oil, sugar, and pineapple powder were collected from the Wolkite Town local market. All raw materials were manually sorted using handpicking to remove the undesirable damaged seeds and foreign materials like nails, nuts, or discolored seeds. The cleaned peanut, chickpea, and maize were soaked in tap water: the chickpea was soaked for 12 h with draining every 4 h, the peanut seed was soaked for 6 h, and the maize grain was soaked for 12 h with draining every 6 h. After soaking, all the ingredients were dried in sunlight until they became crispy. Each dried ingredient was then roasted in a roaster at 150°C for 10 min. Milling was carried out using a hammer mill disc to convert the ingredients into fine flour. The flours were then mixed in the predetermined proportions and packed into polyethylene plastic bags for further laboratory analysis (Nane et al. 2020).

### 2.4 Preparation of Orange‐Flesh Sweet Potato (OFSP) Flour Preparation

The OFSP was sorted, then peeled and washed using tap water. After washing, the OFSP was sliced using a slicer into small pieces (chips) 2–5 mm thick and dried using a cabinet dryer (LEEC Ltd., Serial No. 3114) for 8 h at 65°C, according to the procedure detailed by Dibari et al. (2013). Then, the OFSP chips were crushed into flour using a hammer mill disc (Henan Allways Machinery Co., Limited) and stored in a sealed polyethylene plastic bag before further analysis.

### 2.5 Product Formulation

The proportions of the therapeutic food ingredients were determined using D‐optimal mixture design software to formulate the flour blend (Gemede 2020). Eight combinations of home‐based therapeutic food formulations—peanut (P), chickpea (C), maize (M), and OFSP (PCMOFSP^1^, PCMOFSP^2^, PCMOFSP^3^, PCMOFSP^4^, PCMOFSP^5^, PCMOFSP^6^, PCMOFSP^7^, and PCMOFSP^8^)—were developed, each with varying ingredient proportions (Table 1). The nutrient content of eight formulations was based on the composition designed to align with the recommended daily allowance for children aged 6 to 59 months (Abida Faiz et al. 2024).

**TABLE 1:** Percentage combinations obtained by D‐optimal design for the eight formulations of flour.

Sample codes	Peanut (%)	Chickpea (%)	Maize (%)	OFSP (%)	Pineapple powder (%)	Sugar (%)	Sunflower oil (%)
PCMOFSP^1^	44.00	11.00	7.00	12.00	4.00	12.00	10.00
PCMOFSP^2^	45.20	9.50	10.80	8.50	4.00	12.00	10.00
PCMOFSP^3^	47.00	8.00	8.20	10.80	4.00	12.00	10.00
PCMOFSP^4^	40.50	10.50	12.70	10.30	4.00	12.00	10.00
PCMOFSP^5^	42.00	6.50	10.17	15.33	4.00	12.00	10.00
PCMOFSP^6^	33.50	12.60	5.50	11.40	4.00	12.00	10.00
PCMOFSP^7^	36.50	13.53	11.67	12.30	4.00	12.00	10.00
PCMOFSP^8^	31.30	14.50	16.20	12.00	4.00	12.00	10.00

^1^ Peanut: 44.00%, Chickpea: 11.00%, Maize: 7.00%, Orange flesh sweet potato: 12.00%.

^2^ Peanut: 45.20%, Chickpea: 9.50%, Maize: 10.80%, Orange flesh sweet potato: 8.50%.

^3^ Peanut: 47.00%, Chickpea: 8.00%, Maize: 8.20%, Orange flesh sweet potato: 10.80%.

^4^ Peanut: 40.50%, Chickpea: 10.50%, Maize: 12.70%, Orange flesh sweet potato: 10.30%.

^5^ Peanut: 42.00%, Chickpea: 6.50%, Maize: 10.17%, Orange flesh sweet potato: 15.33%.

^6^ Peanut: 33.50%, Chickpea: 12.60%, Maize: 5.50%, Orange flesh sweet potato: 11.40%.

^7^ Peanut: 36.50%, Chickpea: 13.53%, Maize: 11.67%, Orange flesh sweet potato: 12.30%.

^8^ Peanut: 31.30%, Chickpea: 14.50%, Maize: 16.20%, Orange flesh sweet potato: 12.00%.

### 2.6 Nutrient Analysis

The preparation of all raw materials, development and testing of home‐based therapeutic food, and sensory acceptability tests were carried out at Wolkite University, Department of Food Process Engineering Laboratories, College of Engineering and Technology. The School of Nutrition, Food Science, and Technology Laboratories at Hawassa University was employed to conduct the experiments involving physicochemicals, while the nutritional and antinutritional profile of the product was analyzed at the Ethiopian Health and Nutrition Research Institute (EHNRI), Addis Ababa.

### 2.7 Determination of Physicochemical Properties

#### 2.7.1 Water Activity and pH Meter

The measuring of water activity of the sample was determined by a water activity meter, model number (HD‐3A, NanBei, China). Using a measuring balance, dissolve 10 g of the sample in a beaker filled with 30 mL of distilled water to form a slurry; then it was allowed to stand for 10 min with constant stirring. The pH was measured using a pH meter, model number (BANTE Multipara meter/China), according to official methods (AOAC 2023).

#### 2.7.2 Color

The measuring of the color of the sample was determined by the CIE L* a* b* color space system based on the tristimulus value. The lightness (*L*), redness (+Ve *a*), yellowness (+ve *b*), and the magnitude of total color difference values were measured by placing the sample on the port of the color reader using model number (CR‐10, Konica Minolta, Japan) as *L*, *a*, and *b* values. A positive value of a* indicates the magnitude of the reddish component, while its negative value shows that of the greenish component. A positive value for b* shows a yellowish component, while its negative indicates the bluish component. The L*, a*, and b* values were recorded as (Wodajo and Emire 2022).

### 2.8 Determination of Antinutrient Content

#### 2.8.1 Tannin Content

The tannin content was measured according to a method modified by Dibari et al. (2013) using catechin as the tannin standard. Approximately 1.0 g of each treatment sample was weighed in triplicates in a screw cap test tube and extracted with 10 mL of 1% HCl in methanol for 24 h at room temperature with mechanical shaking. After 24 h of shaking, the solution was centrifuged at 1000 rpm for 5 min. Mix 1 mL of supernatant with 5 mL of vanillin–HCl reagent (prepared by combining an equal volume of 8% concentrated HCl in methanol and 4% vanillin in methanol). The D‐catechin was used as a standard for tannin determination. 0.01 g of D‐catechin was measured and dissolved in 50 mL of 1% HCl, which served as a stock solution. A 0, 0.2, 0.4, 0.6, 0.8, and 1 mL of stock solution were taken in a test tube, and the volume of each test tube was regulated to 1 mL with 1% HCl in methanol. To each test tube, add 5 mL of vanillin–HCl reagent. After 20 min, the absorbance of the sample solutions and the standard solution were evaluated at 500 nm (Perkin Elmer Lambda 950 UV/Vis/NIR, UK), zeroing the spectrophotometer by using distilled water, and the calibration curve was formed from the series of standard solutions as absorbance versus concentration, and the slope and intercept were used for calculation.

#### 2.8.2 Phytate Content

The phytate content in the sample was measured according to the methods described in Dibari et al. (2013). Extract 100 mg of the sample with 10 mL of 0.2 N HCl in a mechanical shaker for 1 h at room temperature. Centrifuge the extract at 1006 x g (*r* = 10cm) for 30 minutes. The clarified supernatant was used for phytate content estimation. Add 1 mL of Wade reagent (containing a 0.03% solution of FeCl_3_. 6H_2_O and 0.3% of sulfosalicylic acid in water) to 3 mL of the sample solution (supernatant) and vortex for 5 s. Absorption readings at 500 nm (Perkin Elmer Lambda 950 UV/Vis/NIR, UK) were taken against an 1188 D. WODAJO AND S. A. EMIRE blank solution (3 mL extract solution mixed with 2 mL of 2.4% HCl). The sodium salt of phytic acid (5–36 mg/mL) was used as a standard to construct the calibration curve. The phytate concentration was determined from a standard curve, and results were expressed as phytic acid in mg per 100 g dry matter.

### 2.9 Determination of Proximate Composition

Proximate composition (moisture, ash, fiber, protein, and fat) was determined using AOAC International standards (AOAC 2020). Moisture content was assessed according to AOAC (2020) Official Method 925.10, in a dry oven (Model 10‐ D1391/AD, SCA) at 105°C for 12 h until constant weight. The ash content (%) was determined following AOAC Official Method 923.03. This involved incinerating the sample at 550°C for 6 h in a NATEK MKF‐07 muffle furnace (Turkey) and calculating the percentage based on the mass difference before and after incineration. Crude fiber content (% CF) was analyzed following AOAC Official Method 962.09. This involved sequential acid and alkali hydrolysis performed using a BXB‐06 hydrolysis system (Guangzhou, China) to quantify the fiber fraction. The crude protein content (% CP) was analyzed using the Kjeldahl method (AOAC 984.13), where nitrogen levels (N) were quantified with a (Hanon K1160) automatic Kjeldahl analyzer. The percentage of crude protein was then calculated by multiplying the measured nitrogen content by the standard nitrogen‐to‐protein conversion factor of 6.25. Fat content was analyzed using the Soxhlet extraction method (AOAC 920.39), with petroleum ether serving as the solvent to isolate and quantify lipids. Carbohydrate content was calculated by difference including fiber: CHO % = 100‐(moisture content % + crude protein % + fat % + fiber % + ash %). The determination of energy content was done by using Atwater's conversion factor: 4 kcal/g for carbohydrates, 4 kcal/g for protein, and 9 kcal/g for fat (Nane et al. 2020).

### 2.10 Determination of Mineral Content

The calcium content was determined using the AOAC official Method 923.03 EDTA titration (AOAC 2020). The iron, zinc, potassium, and phosphorus contents were measured using an atomic absorption/emission spectrophotometer (Nane et al. 2020).

### 2.11 Sensory Acceptability Test

The panelists were trained on the use of sensory evaluation procedures and the meaning of the descriptive terms used. The panelists for the test checked their health status and excluded individuals who smoke, have allergies, and have smell sensitivity. Those who volunteered to be tested were included. As mothers/caregivers are primary decision‐makers in child‐feeding practices, they were instructed to taste and evaluate the samples on evaluation sheets and asked to sign a consent form before undergoing the testing. A total of 50 untrained female panelists participated in this study, and the age of the females was above 25 years. Just before the test session, orientation was given to the panelists on the procedure of sensory evaluation. The samples from each selected product were arranged in random order on white plates and served to the panelists. Samples were ordered on the table without providing any information to the panelists. A bottle of tap water with white plastic cups was given to rinse their mouths after each test. They used their observations and sense organs to make decisions and interpret the nature of a sample. Sensory attributes measured were color, aroma, taste, texture, and overall acceptability using a five‐point hedonic scale, where 1‐dislike extremely; 2‐dislike slightly; 3‐neither like nor dislike; 4‐like slightly; 5‐like extremely (Wodajo and Emire 2022). The use of mothers instead of the target recipient infant was necessary because of their ability to evaluate objectively the sensory characteristics of the formulations to the interest of their children. After tasting each coded sample, they rinsed their mouths and spat it out into a receiving pot before moving on to the next sample. The results were recorded and analyzed to determine the significant variations of sensory attributes of the product's average scores.

### 2.12 Rate of Change in Acid and Peroxide Value

The acid value was determined according to the method (Jenfa et al. 2024).

Peroxide value was determined according to a method (Chiedu et al. 2023).

### 2.13 Microbial Load Analysis

#### 2.13.1 Total Plate Count (TPC)

A 10 g sample was aseptically weighed into 90 mL of sterile salt peptone solution (XPS) containing 0.1% peptone and 0.8% sodium chloride with pH adjusted to 7.2 and homogenized in the Stomacher (model 4001, Seward Medical) for 30 s at normal speed. This provided 10^−1^ dilution. This was vortexed for about 2 min to ensure uniform mixing. Using a sterile pipette, 1 mL of the ^−1^10‐fold dilution was pipetted into 9 mL of sterile salt peptone water to obtain 10–^2^ dilution. This procedure was repeated for 10^−3^, 10^−4^, 10^−5^, and 10^−6^ dilutions. Further, from the appropriate tenfold serial dilution, a 1 mL aliquot of each dilution (10^−1^, 10^−2^, 10^−3^, 10^−4^, 10^−5^, and 10^−6^) was inoculated into sterile Petri dish plates, and the appropriate media was added for enumeration and isolation. After appropriate incubation, dilutions with 30–300 colonies were selected and counted. The number of colony‐forming units per mL (cfu/mL) of food was calculated by multiplying the number of bacteria by the dilution. All analyses were done in triplicate for the reliability of results.

### 2.14 Statistical Analysis

All data were analyzed using a one‐way analysis of variance (ANOVA) model with Statistical Analysis System (SAS) software, version 9.4 (SAS Institute Inc., Cary, North Carolina, USA). Before ANOVA, the normality of residuals was checked using the Shapiro–Wilk test (*p* > 0.05), ensuring compliance with ANOVA assumptions. Significant differences among treatment means were identified using Tukey's Honestly Significant Difference (HSD) test at a 5% significance level (*p* < 0.05).

## 3 Results and Discussion

### 3.1 Physical Properties

#### 3.1.1 Water Activities and pH Meter

As shown in Table 2, the water activity data indicate that the processing techniques have minimal to no impact on the shelf life of the therapeutic food products, with significant differences observed between the treatments (*p* < 0.05). The measured values for the sample are detailed in the table. The pH values of the food samples ranged from 3.95 to 4.35, with significant differences noted among treatments (*p* < 0.05). Specifically, the pH value levels, which reflect the acidity or alkalinity of the samples, ranged from 3.95 for peanut (P), chickpea (C), maize (M), and OFSP (PCMOFSP^8^) to 4.35 for PCMOFSP^7^, indicating relatively low acidity. When comparing foods with higher acidity (pH < 4) to those formulated to maintain lower acidity, the former are less suitable for applications such as baking and pastry, where a neutral or lower acidity is generally preferred (Wodajo and Emire 2022). According to Nezif (2020), the pH values of the sample in an aqueous suspension are critical, as they influence key functional properties such as solubility and emulsion stability.

**TABLE 2:** Physical properties (water activity, pH, and color) of home‐based paste food per 100 g.

Sample codes	Water activity	pH meter	L*	a*	b*	C*	*H*
PCMOFSP^1^	0.69 ± 0.00^c^	4.24 ± 0.05^dc^	52.27 ± 0.91^c^	10.49 ± 1.04^a^	6.39 ± 0.05^f^	11.48 ± 0.56^d^	34.37 ± 0.61^g^
PCMOFSP^2^	0.71 ± 0.01^ba^	4.13 ± 0.04^e^	49.61 ± 1.05^dc^	5.89 ± 1.13^cb^	8.62 ± 0.57^e^	10.76 ± 0.79^d^	55.74 ± 0.89^d^
PCMOFSP^3^	0.70 ± 0.01^bc^	4.23 ± 0.04^d^	48.05 ± 1.57^**d**^	4.63 ± 1.01^cd^	5.42 ± 0.57^g^	6.82 ± 0.89^e^	49.76 ± 0.69^e^
PCMOFSP^4^	0.69 ± 0.01^b**c**^	4.23 ± 0.04^d^	66.01 ± 1.53^a^	5.67 ± 0.99^cbd^	27.56 ± 0.71^a^	27.88 ± 0.88^a^	78.54 ± 0.54^a^
PCMOFSP^5^	0.72 ± 0.02^a^	4.31 ± 0.04^ba^	65.72 ± 0.92^a^	6.84 ± 0.91^**b**^	25.79 ± 0.80^b^	26.79 ± 0.93^a^	75.54 ± 0.69^b^
PCMOFSP^6^	0.71 ± 0.00^ba^	4.29 ± 0.02^bc^	59.71 ± 1.78^b^	5.33 ± 0.66^cbd^	20.78 ± 0.75^c^	21.67 ± 0.78^b^	76.64 ± 0.82^b^
PCMOFSP^7^	0.70 ± 0.08^ba^	4.35 ± 0.02^a^	52.70 ± 1.53^c^	5.06 ± 0.41^cd^	12.54 ± 0.53^d^	13.64 ± 0.78^c^	69.66 ± 0.74^c^
PCMOFSP^8^	0.48 ± 0.01^d^	3.95 ± 0.02^f^	42.91 ± 3.18^e^	4.19 ± 0.49^d^	3.53 ± 0.54^h^	5.49 ± 0.66^f^	41.97 ± 0.65^f^
LSD	**0.02**.	**0.36**	**3.12**	**1.61**	**0.32**	**1.28**	**1.23**
CV	**1.30**	**0.79**	**3.25**	**9.25**	**1.33**	**4.68**	**1.16**

*Note:* Mean different superscripts within the same column are significantly different (*p* < 0.05).

Abbreviations: a*, redness; b*, yellow; *c*, chroma; CV, coefficient of variation; *h*, hue angle; L*, whiteness; LSD, least significant difference.

^1^ Peanut: 44.00%, Chickpea: 11.00%, Maize: 7.00%, Orange flesh sweet potato: 12.00%.

^2^ Peanut: 45.20%, Chickpea: 9.50%, Maize: 10.80%, Orange flesh sweet potato: 8.50%.

^3^ Peanut: 47.00%, Chickpea: 8.00%, Maize: 8.20%, Orange flesh sweet potato: 10.80%.

^4^ Peanut: 40.50%, Chickpea: 10.50%, Maize: 12.70%, Orange flesh sweet potato: 10.30%.

^5^ Peanut: 42.00%, Chickpea: 6.50%, Maize: 10.17%, Orange flesh sweet potato: 15.33%.

^6^ Peanut: 33.50%, Chickpea: 12.60%, Maize: 5.50%, Orange flesh sweet potato: 11.40%.

^7^ Peanut: 36.50%, Chickpea: 13.53%, Maize: 11.67%, Orange flesh sweet potato: 12.30%.

^8^ Peanut: 31.30%, Chickpea: 14.50%, Maize: 16.20%, Orange flesh sweet potato: 12.00%.

#### 3.1.2 Color

Each peanut (P), chickpea (C), maize (M), and OFSP (PCMOFSP) food sample's lightness was represented by its L* value, where 0 indicates black and 100 indicates white. The measured color values for the samples are shown in Table 2, with L* values ranging from 42.91 to 66.01. Significant differences were observed among the treatments (*p* < 0.05). PCMOFSP^3^ sample has indicating signficance differnt from compare other samples. The lightness index (L*) reflects brightness on a scale from black to white, with PCMOFSP^4^ exhibiting the highest value (66.01) and PCMOFSP^8^ the lowest (42.91). The lower L* value of PCMOFSP^8^ may be due to a reduced proportion of lighter‐colored ingredients, resulting in a darker or more intense color. The measured a* color values of the samples are displayed in Table 2, indicating the red‐green spectrum, ranging from 4.19 to 10.49, with PCMOFSP^1^ having the highest value (10.49). This increased redness is likely due to the grain's red pigmentation, which becomes mixed into the flour (Sharanagat et al. 2024). PCMOFSP^8^ showed the lowest a* value (4.19). The b* values ranged from 3.53 to 27.56, with significant differences among the treatments (*p* < 0.05). PCMOFSP^4^ showed the highest b* value (27.56), while PCMOFSP^8^ had the lowest (3.53). The chroma values for the samples, as shown in Table 2, showed significant differences among the treatments (*p* < 0.05). The sample code PCMOFSP^4^ recorded the highest chroma value (27.88), while sample code PCMOFSP^8^ had the lowest (5.49). The hue angle values of the samples, as presented in Table 2, ranged from 34.37° to 78.54°. Sample PCMOFSP^4^ showed the highest hue angle (78.54°), while sample PCMOFSP^8^ had the lowest (34.37°). Consistent with Abida Faiz et al. (2024), our results suggest that peel fragment pigments, inadvertently incorporated during sample preparation, can alter hue values, thereby affecting the sample's perceived redness or yellowness. Similar findings indicate that pigments from peel fragments introduced during sample preparation could influence hue values. These pigments impact the redness or yellowness of the sample (Singh et al. 2023). Additionally, factors such as contaminants and milling conditions can affect flour color (Haile et al. 2016). Color changes also reflect the extent of heating and pigment degradation during starch extraction, as well as browning reactions like caramelization and the Maillard reaction (Tortoe et al. 2017). Such color shifts can impact not only appearance but also nutritional value, as certain pigments, like carotenoids linked to essential nutrients, degrade during processing (Vijay 2018). The concentration of color observed in this study was lower compared to those previously reported by AlOudat et al. (2021) and this could be due to the different processing methods involved in the production of the current home‐based therapeutic food.

### 3.2 Anti‐Nutrient Content, Peroxide, and Acid Value

Antinutrients are compounds present in food that inhibit the function of one or more nutrients, lowering their digestibility and the body's ability to absorb them (Samtiya et al. 2020). This often occurs when antinutrients bind to nutrients, forming complexes that restrict absorption (Yegrem 2021). Substances such as tannins and phytates, classified as antinutritional factors, notably diminish nutrient bioavailability by disrupting critical processes like digestion, absorption, or the body's utilization of essential nutrients (Salim et al. 2023). Research indicates that tannins bind to proteins, whether enzymatic or non‐enzymatic, to form tannin‐protein complexes, impairing protein digestion and solubility. This inhibition likely stems from either the inactivation of digestive enzymes or reduced enzymatic accessibility to substrate proteins post‐complex formation. Additionally, tannins diminish the bioavailability of vitamins (A, B‐group, and C) and minerals such as iron, zinc, iodine, phosphorus, and magnesium (Gupta et al. 2015). Notably, food‐processing techniques like roasting, smoking, and germination can significantly reduce tannin content (Salim et al. 2023). Phytate (inositol hexakisphosphate), a phosphorus‐rich compound, acts as an antinutrient by binding to essential dietary minerals, thereby impeding their absorption. Its molecular structure, characterized by a dense array of negatively charged phosphate groups, enables the formation of stable complexes with mineral ions, rendering them unavailable for intestinal uptake and reducing bioavailability (Cichon et al. 2023). This interaction poses significant nutritional concerns, as phytate notably inhibits the absorption of key minerals such as zinc, iron, calcium, magnesium, manganese, and copper (Sangeetha et al. 2022).

#### 3.2.1 Tannin Content

As shown in Table 3, tannin content ranges from 1.75 mg/g to 2.39 mg/g, with significant differences observed among the treatments (*p* < 0.05). The PCMOFSP^5^, PCMOFSP^1^, PCMOFSP^4^, PCMOFSP^7^, signifcance different among other samples (*p* < 0.05). PCMOFSP^5^ has the highest tannin content (2.39 mg/g), and PCMOFSP^4^ has the lowest (1.75 mg/g). These tannin content values fall well below the 10–60 mg/g range reported to be potentially harmful to human health (Ojo 2022). Excessive tannin intake above 10 mg/g, if consumed frequently, may lead to nutrient deficiencies by hindering the absorption of protein and essential minerals, such as iron. The level of tannins in PCMOFSP^5^ was close to those reported by Nane et al. (2020) for sorghum and OFSP flour. Condensed tannins, even at a low level, possess an astringent taste, which contributes to a decreased intake of foods (Cosme et al. 2025). Condensed tannins inhibit enzymatic digestion of protein by forming complexes with large quantities of proteins, but this only occurs when present in high amounts (Ojo 2022). In addition, plant‐source foods, in particular legumes or a blend of cereals and legumes, contain substantial amounts of tannins, which limit the absorption of some minerals (Mohammadi 2024). The number of tannins and other naturally occurring toxins in supplementary foods must be reduced through appropriate food‐processing methods, such as roasting, soaking, germination, malting, and fermentation (Wodajo and Emire 2022).

**TABLE 3:** Antinutrient content, and (peroxide value, acid value) of home‐based paste food/100 g of edible portion.

Sample codes	Tannin content (mg/100 g)	Phytate content (mg/100 g)	Acid value (mg)	Peroxide value (meq)
PCMOFSP^1^	2.11 ± 0.17^bac^	0.67 ± 0.03^a^	0.73 ± 0.04^c^	5.06 ± 0.85^a^
PCMOFSP^2^	1.92 ± 0.13^bdc^	0.59 ± 0.03^b^	0.86 ± 0.05^a^	4.44 ± 0.54^bac^
PCMOFSP^3^	1.77 ± 0.11^dc^	0.51 ± 0.03^c^	0.76 ± 0.09^bc^	4.65 ± 0.76^ba^
PCMOFSP^4^	1.75 ± 0.26^d^	0.47 ± 0.01^c^	0.91 ± 0.02^a^	3.92 ± 0.24^dc^
PCMOFSP^5^	2.39 ± 0.09^a^	0.40 ± 0.00^d^	0.92 ± 0.06^a^	4.39 ± 0.52^bc^
PCMOFSP^6^	1.88 ± 0.19^dc^	0.38 ± 0.01e^d^	0.62 ± 0.04^d^	3.69 ± 0.28^d^
PCMOFSP^7^	2.25 ± 0.25^ba^	0.35 ± 0.01^e^	0.85 ± 0.01^ba^	3.83 ± 0.32^dc^
PCMOFSP^8^	1.78 ± 0.00^ba^	0.29 ± 0.06^f^	0.63 ± 0.05^d^	3.83 ± 0.40^dc^
LSD	**0.35**	**0.05**	**0.09**	**5.34**
CV	**10.00**	**5.81**	**6.62**	**8.34**

*Note:* Mean different superscripts within the same column are significantly different (*p* < 0.05).

Abbreviations: CV, coefficient of variation; LSD, least significant difference; meq, milliequivalent; Mg, milligram.

^1^ Peanut: 44.00%, Chickpea: 11.00%, Maize: 7.00%, Orange flesh sweet potato: 12.00%.

^2^ Peanut: 45.20%, Chickpea: 9.50%, Maize: 10.80%, Orange flesh sweet potato: 8.50%.

^3^ Peanut: 47.00%, Chickpea: 8.00%, Maize: 8.20%, Orange flesh sweet potato: 10.80%.

^4^ Peanut: 40.50%, Chickpea: 10.50%, Maize: 12.70%, Orange flesh sweet potato: 10.30%.

^5^ Peanut: 42.00%, Chickpea: 6.50%, Maize: 10.17%, Orange flesh sweet potato: 15.33%.

^6^ Peanut: 33.50%, Chickpea: 12.60%, Maize: 5.50%, Orange flesh sweet potato: 11.40%.

^7^ Peanut: 36.50%, Chickpea: 13.53%, Maize: 11.67%, Orange flesh sweet potato: 12.30%.

^8^ Peanut: 31.30%, Chickpea: 14.50%, Maize: 16.20%, Orange flesh sweet potato: 12.00%.

#### 3.2.2 Phytate Content

As shown in Table 3, phytate content ranged from 0.29 mg/g to 0.67 mg/g, with there being significant differences observed among the treatments (*p* < 0.05). PCMOFSP^1^ had the highest phytate content (0.67 mg/g), while PCMOFSP^8^ had the lowest (0.29 mg/g). Composite flour blends using peanuts and chickpeas contained higher phytate content than other ingredients. According to (Duguma et al. 2021), phytate levels should be reduced to below 200 mg/100 g to mitigate their negative effects on mineral absorption. The study indicates that most samples had phytate levels within an acceptable range for minimizing this impact (Nissar et al. 2017). The high levels of phytate in PCMOFSP^1^ could be due to high levels of phytates in peanuts and chickpeas (Nissar et al. 2017). A diet containing substantial amounts of phytate reduces the bioavailability of minerals, particularly iron, calcium, magnesium, and zinc, which are important in the treatment of MAM in children (Gupta et al. 2015).

#### 3.2.3 Acid Value

The shelf life of the home‐based food was monitored over 8 weeks. The product was packaged in double polyethylene plastic bags, and measurements of acid and peroxide values were taken at the start and then weekly at room temperature to track stability. Over the 8‐week storage period using polyethylene bags, the sample acid value gradually increased, reflecting a rise in free fatty acids due to potential oxidative degradation. Starting at an initial acid value (AV) of 0.62 mg KOH/g, it reached 0.92 mg KOH/g by day 56. This rise indicates a slow breakdown of fat, though the acid value (AV) remains within the safe limit for edible oils, such as sunflower oil, which typically requires an acid value (AV) of < 2 mg KOH/g to ensure freshness and safety, according to standard guidelines and studies on fat oxidation in storage. Generally, a product's acid value reflects its free fatty acid content, signifying the extent of hydrolytic breakdown of triacylglycerols, which determines the fat or oil acidity (Medeiros Vicentini‐Polette et al. 2021). Acidity is a key indicator of a product's quality and authenticity, as changes often result from inadequate handling or preservation of fats. Such increases in fatty acids are well documented in studies on food storage, where both packaging type and storage duration significantly affect fatty acid levels (Muzeza et al. 2023). In this study, the acid values of the sample were lower than those of food lends used for the management of MAM and close to the recommended values for acid value (Cichon et al. 2023).

#### 3.2.4 Peroxide Value

The peroxide value (PV) of the samples stored in polyethylene bags exhibited a gradual increase over 8 weeks, with weekly measurements showing a consistent rise. Initially, the peroxide value (PV) was 3.69 meq/kg and reached 5.06 meq/kg by day 56, indicating ongoing oxidative changes in the fats, likely due to continued exposure to oxygen in the storage environment. Although the peroxide value (PV) increased, it remained within acceptable limits, as the typical PV for fresh sunflower oil is under 10 meq/kg (Bustani and Soni 2023). Since elevated peroxide value (PV) levels are linked to rancidity and a reduction in nutritional quality, monitoring peroxide levels is essential for assessing lipid oxidation, which plays a key role in determining the shelf life and sensory quality (Geng et al. 2023).

### 3.3 Proximate Composition

Home‐based therapeutic foods present potential benefits over current treatments for MAM and address limitations in sustainability, affordability, accessibility, and cost‐effectiveness using locally available ingredients at the household level. The existing treatment of MAM primarily confines supplementary feeding programs (SFPs) to districts identified as chronically food insecure (Nane et al. 2020).

#### 3.3.1 Moisture Content

The measured moisture content of the sample is presented in Table 4. The moisture content values ranged from 4.56% to 8.79%. There were significant differences among the treatments (*p* < 0.05). The PCMOFSP^4^ (8.79) and PCMOFSP^7^(8.49) samples are signifacnt different compare to other sample. PCMOFSP^4^ had the highest value (8.79%), and PCMOFSP^2^ had the lowest value (4.56%). This study showed that moisture content remained consistent across the eight components, showing significant variation. Moisture content in food samples serves as a key indicator of stability, impacting product appearance, quality, and yield (Shanker et al. 2019). Understanding moisture content is crucial for predicting how foods will behave during processing steps like mixing, drying, storage, and packaging (Akinmoladun et al. 2023). Assessing moisture content is essential to determine whether the food is suitable for consumption (Maru et al. 2024). The moisture content affects the physical and chemical aspects of the food, which relates to freshness and stability (Tapia et al. 2020). The storage of the food for a long period and the moisture content determine the actual quality of the food before consumption and the subsequent processing in the food sector by the food producers (Bayala‐Yaї et al. 2024). In this study, the moisture content of the formulated sample is at a low level, and this might be due to the drying methods (Nezif 2020). The low‐moisture foods are not easily susceptible to microbial attack and have a longer shelf life (Brixi 2018). In a similar study, the moisture content is within the recommended level for the proper storage of dehydrated foodstuff (FAO 2017). This is an indication that the samples would have a longer shelf life during storage (Wamunga and Wamunga 2017). Monitoring the moisture content in foods and food products is crucial because high moisture content can reduce shelf life by increasing microbial degradation activity, resulting in bad odor and unacceptable taste of the product (Borg et al. 2020).

**TABLE 4:** Proximate composition (%) and energy content (Kcal/100 g) of eight formulated foods.

Sample codes	Moisture content (%)	Ash content (%)	Fat content (%)	Fiber content (%)	Protein content (%)	CHO (%)	Energy content (Kcal/100 g)
PCMOFSP^1^	5.85 ± 0.85^bc^	1.79 ± 0.33^c^	33.03 ± 0.61^a^	2.73 ± 0.25^ba^	13.91 ± 0.05^a^	42.71 ± 1.29^cb^	524.53 ± 7.07^ba^
PCMOFSP^2^	4.56 ± 1.29^c^	2.58 ± 1.17^b^	33.79 ± 0.22^a^	2.65 ± 0.44^ba^	13.51 ± 0.05^b^	42.91 ± 2.25^cb^	529.81 ± 10.60^a^
PCMOFSP^3^	7.48 ± 0.63^ba^	1.99 ± 0.02^a^	34.62 ± 0.15^a^	2.63 ± 0.31^ba^	12.42 ± 0.02^c^	41.44 ± 0.49^cb^	527.04 ± 3.30^ba^
PCMOFSP^4^	8.79 ± 1.18^a^	1.89 ± 0.03^d^	34.14 ± 0.26^a^	2.39 ± 0.53^bc^	12.40 ± 0.01^c^	40.37 ± 1.02^c^	518.34 ± 6.07^bac^
PCMOFSP^5^	6.51 ± 0.53^bac^	2.04 ± 0.05^d^	31.78 ± 6.65^ba^	2.49 ± 0.10^b^	12.01 ± 0.00^d^	45.29 ± 5.76^b^	515.18 ± 3.42^bac^
PCMOFSP^6^	7.11 ± 2.19^ba^	1.88 ± 0.35^d^	31.61 ± 0.21^ba^	2.63 ± 0.06^ba^	11.49 ± 0.02^e^	45.73 ± 2.28^b^	511.60 ± 0.62 ^bac^
PCMOFSP^7^	8.49 ± 0.56^a^	2.22 ± 0.11^c^	30.65 ± 0.59^ba^	3.03 ± 0.32^a^	11.04 ± 0.09^f^	44.12 ± 0.34^cb^	498.31 ± 5.26^c^
PCMOFSP^8^	5.35 ± 2.14^bc^	2.13 ± 0.21^c^	28.06 ± 0.87^a^	1.93 ± 0.25^c^	10.03 ± 0.02^g^	52.50 ± 2.66^a^	502.64 ± 4.79^bc^
LSD	**2.46**	**0.82**	**4.11**	**0.31**	**4.63**	**0.43**	**5.68**
CV	**9.9**	**8.56**	**7.28**	**10.63**	**0.34**	**5.96**	**2.84**

*Note:* Mean different superscripts within the same column are significantly different (*p* < 0.05).

Abbreviations: CHO, carbohydrate; CV, coefficient of variation; LSD, least significant difference.

^1^ Peanut: 44.00%, Chickpea: 11.00%, Maize: 7.00%, Orange flesh sweet potato: 12.00%.

^2^ Peanut: 45.20%, Chickpea: 9.50%, Maize: 10.80%, Orange flesh sweet potato: 8.50%.

^3^ Peanut: 47.00%, Chickpea: 8.00%, Maize: 8.20%, Orange flesh sweet potato: 10.80%.

^4^ Peanut: 40.50%, Chickpea: 10.50%, Maize: 12.70%, Orange flesh sweet potato: 10.30%.

^5^ Peanut: 42.00%, Chickpea: 6.50%, Maize: 10.17%, Orange flesh sweet potato: 15.33%.

^6^ Peanut: 33.50%, Chickpea: 12.60%, Maize: 5.50%, Orange flesh sweet potato: 11.40%.

^7^ Peanut: 36.50%, Chickpea: 13.53%, Maize: 11.67%, Orange flesh sweet potato: 12.30%.

^8^ Peanut: 31.30%, Chickpea: 14.50%, Maize: 16.20%, Orange flesh sweet potato: 12.00%.

#### 3.3.2 Ash Content

The total ash content of the samples is shown in Table 4, with values ranging from 1.79% in PCMOFSP^1^ to 2.58% in PCMOFSP^2^. Signifcance difference PCMOFSP^7^(2.22) and PCMOFSP^8^ (2.13). Among these, PCMOFSP^2^ showed the highest ash content value of 2.58%, while PCMOFSP^1^ had the lowest at 1.79%. Significant differences were observed in the ash content across all therapeutic food samples (*p* < 0.05). This study indicated that the total ash content was higher in the product formulated with a high proportion of chickpea flour (Forsido et al. 2019). This could be attributed to the naturally high ash content in chickpeas (Arogundade et al. 2023), which substantially contributes to the elevated ash levels in these chickpea‐based blended therapeutic food products. This suggests that increasing the proportion of chickpeas relative to maize can enhance the ash (mineral) content of the infant food (Dibari et al. 2013). A previous study supports this finding, showing that ash content in blended infant foods rises with the addition of chickpea blend flour (Haile et al. 2016). Generally, the ash content in home‐prepared therapeutic foods depends on the mineral content of the ingredients used (Nezif 2020). To meet infants' mineral needs, a range of mineral‐rich, home‐based therapeutic foods should be provided (Schoonees et al. 2019). The ash contents observed in this study were lower than 2.37%–5.67% reported for the composite flour of wheat, bambara nut, and OFSP (Ubbor et al. 2022).

#### 3.3.3 Fat Content

The measured fat content of the samples is presented in Table 4. The fat content across the eight therapeutic food samples ranged from 28.06% in PCMOFSP^8^ to 34.62% in PCMOFSP^3^. The lowest fat content was observed in PCMOFSP^8^ (28.06%), while PCMOFSP^3^ had the highest (34.62%). PCMOFSP^8^ showed a significantly lower fat content compared to the other samples, while PCMOP^3^ had a significantly higher fat content than the rest of the treatments. The fat content in all therapeutic foods falls within the recommended range for therapeutic supplements for MAM (Cichon et al. 2023). The highest fat content in PCMOFSP^4^ may be due to its formulation, which includes a substantial proportion of peanuts, known for their high crude fat and oil content (Nane et al. 2022). Children with MAM have increased energy needs, requiring a diet rich in fats to support both energy intake and the absorption of fat‐soluble vitamins like A and E (Borg et al. 2020). Thus, therapeutic foods must contain sufficient fat to provide the necessary energy for malnourished children, as supported by findings from other studies (Bayala‐Yaї et al. 2024). A diet too low in fat can make it challenging for children to obtain enough energy due to its bulk (Monnard and Fleith 2021). The recommended daily allowance for fat in complementary foods for infants and young children is 30%–40%, equivalent to 13.33–20.00 g/100 g of energy (Abeshu et al. 2016). To ensure adequate calories from fat, adding fat during food formulation is advisable (Samuel et al. 2019). This finding aligns with the report by (Bayala‐Yaї et al. 2024), which highlights that sweet potatoes are a rich source of ash.

#### 3.3.4 Fiber Content

The measured fiber content of the sample is presented in Table 4. The fiber content across the eight therapeutic food samples ranged from 1.93% in PCMOFSP^8^ to 3.03% in PCMOFSP^7^. PCMOFSP^7^ exhibited the highest fiber content value at 3.03%, while PCMOFSP^8^ had the lowest value at 1.93%. The fiber content of the PCMOFSP sample ranged from 1.93% to 3.03% and exhibited significantly higher fiber levels than the other samples. This higher fiber content in PCMOFSP^7^ can be attributed to its greater proportion of chickpeas, which are known for their high dietary fiber content, making them an essential part of the human diet (Begum et al. 2023). The dietary fiber content plays a crucial role in digestion, with soluble fiber content offering prebiotic benefits and insoluble fiber helping to prevent constipation (Nane et al. 2022). While constipation is not a primary concern for malnourished children, it is generally recommended to limit insoluble fiber intake while increasing soluble fiber consumption in children's diets (Lionetti et al. 2023). However, due to limited evidence on the adverse effects of insoluble fiber in children, no specific limits have been established (Santini et al. 2013). Therefore, all products in this study remain within the recommended daily allowance (RDA) for crude fiber (MOH 2019). The fiber contents observed in this study were lower than 2.75%–3.39% reported for complementary foods formulated from maize, pea, and anchote flour (Gemede 2020).

#### 3.3.5 Protein Content

The protein content of the food sample is shown in Table 4. PCMOFSP^1^ exhibited the highest protein content value of 13.91%, while PCMOFSP^8^ had the lowest value of 10.03%. In the PCMOFSP foods in eight formulated therapeutic products, there is a significant difference among the treatments (*p* < 0.05). The protein content of the sample ranged from 10.03% to 13.91%, with PCMOFSP^1^ having the highest protein content at 13.91%, significantly more than the other samples. The protein content across all eight PCMOFSP samples aligns with the recommended values for managing MAM (USAID 2023). The higher protein content in PCMOFSP^1^ likely results from its formulation, which includes a higher proportion of chickpeas and peanuts, both excellent sources of high‐quality protein that contribute substantially to the recommended daily protein intake (Dragičević et al. 2015). Given their high protein content, incorporating chickpeas and peanuts in this therapeutic food may be effective in addressing protein‐energy malnutrition (Brixi 2018). All crude protein values in this study exceed the WHO/UN minimum requirement of 9% (United Nations 2021). Our formulation contains 13.91 g of protein per 100 g, which is lower than the protein levels reported in specialized therapeutic formulations (20.3–22.6 g/100 g) by Nane et al. (2020).

#### 3.3.6 Carbohydrate Content

The measured carbohydrate content of the sample is shown in Table 4. The carbohydrate content of the food samples ranged from 40.37% in PCMOFSP^4^ to 52.5% in PCMOFSP^8^. PCMOFSP^8^ had the highest carbohydrate content at 52.5%, and PCMOFSP^4^ had the lowest at 40.37%. Significant differences in carbohydrate content were observed across the treatment (*p* < 0.05). The total utilizable carbohydrate content ranges from 40.37% to 52.5%. The formulated food product made with maize flour had the highest carbohydrate content, followed by the OFSP blend (Sigh et al. 2018). The sample containing peanut flour had the lowest carbohydrate content, likely due to peanuts being high in protein and relatively low in carbohydrate content. In many developing countries, root crops, tubers, and cereal grains are the primary sources of dietary carbohydrates. Studies by Wodajo and Emire (2022) confirm that maize provides high levels of digestible, small‐sized starch, offering more carbohydrates than many grain cereals. Digestible carbohydrates are crucial for dietary energy in infancy and childhood, supporting essential growth and development.

#### 3.3.7 Energy Content

The energy content of the sample is presented in Table 4, with values ranging from 498.31 to 529.81 Kcal/100 g across the eight treatments. PCMOFSP^2^ exhibited the highest energy content value of 529.81 kcal/100 g, while PCMOFSP^7^ had the lowest value of 498.31 Kcal/100 g, with significant variations observed between the samples. Energy density plays a pivotal role in foods formulated for children with MAM, given their increased energy requirements (Nane et al. 2020). Importantly, all eight PCMOFSP samples surpassed the minimum recommended energy threshold of 380 Kcal/100 g for fortified blended foods (Fiorentino et al. 2018). The energy contents of maize flour were higher than those of chickpea and peanut flour (Temba et al. 2017). This indicates that maize flour could be a major source of energy.

### 3.4 Mineral Contents

#### 3.4.1 Calcium Content

Table 5 presents the mineral content of eight formulated home‐based therapeutic foods that were determined. It has been reported with the potential to prevent type‐2 diabetes (Govender et al. 2021). The mineral content of all samples signifcant difference (*p* < 0.05). The measured calcium content of the sample is shown in Table 5. The calcium content of the samples shows a significantly different outcome among the treatments, ranging from 100.42 mg to 115.51 mg per 100 g. PCMOFSP^3^ had the highest calcium content value of 115.51 mg, while PCMOFSP^5^ had the lowest value of 100.42 mg. However, the calcium content in all the PCMOFSP samples was below the recommended amounts for children with MAM, as outlined by Nane et al. (2020). This pattern can be explained by the elevated calcium levels found in the sorghum, as reported by Golden (2009). According to Gemede (2020), calcium plays essential roles as a structural component of bones and teeth and a regulator of nerve and muscle functions.

**TABLE 5:** Mineral contents (mg/100 g of edible portion) of eight formulated home‐based foods.

Sample codes	Calcium content (mg)	Zinc content (mg)	Iron content (mg)	Potassium content (mg)	Phosphorous content (mg)
PCMOFSP^1^	104.95 ± 5.01^c^	5.36 ± 0.57^cbd^	8.39 ± 0.52^c^	553.95 ± 0.51^dc^	442.54 ± 0.56^e^
PCMOFSP^2^	111.45 ± 0.65^b^	5.63 ± 0.67^cb^	10.22 ± 0.86^ba^	555.44 ± 0.85^c^	444.47 ± 5.6^d^
PCMOFSP^3^	115.51 ± 0.58^a^	5.65 ± 0.58^cb^	9.33 ± 0.62^bc^	550.43 ± 0.79^d^	450.48 ± 0.59^b^
PCMOFSP^4^	107.40 ± 0.81^c^	6.49 ± 0.45^a^	10.58 ± 0.77^a^	556.66 ± 0.69^c^	451.84 ± 0.37^a^
PCMOFSP^5^	100.42 ± 0.71^d^	5.20 ± 0.39^cd^	11.34 ± 0.62^a^	544.15 ± 0.96^e^	447.37 ± 0.64^c^
PCMOFSP^6^	101.03 ± 2.05^d^	5.01 ± 0.09^d^	10.39 ± 0.62^ba^	565.64 ± 6.41^ba^	446.30 ± 0.72^c^
PCMOFSP^7^	111.19 ± 0.89^b^	5.84 ± 0.21^b^	8.98 ± 1.03^c^	569.45 ± 0.99^a^	450.55 ± 1.32^b^
PCMOFSP^8^	105.33 ± 1.10^c^	6.74 ± 0.46^a^	9.37 ± 0.68^bc^	661.57 ± 1.00^b^	444.51 ± 0.68^d^
LSD	**3.07**	**0.59**	**1.16**	**4.09**	**1.29**
CV	**1.64**	**5.91**	**6.76**	**0.42**	**0.17**

*Note:* Mean different superscripts within the same column are significantly different (*p* < 0.05).

Abbreviations: CV, coefficient of variation; LSD, least significant difference; Mg, milligram.

^1^ Peanut: 44.00%, Chickpea: 11.00%, Maize: 7.00%, Orange flesh sweet potato: 12.00%.

^2^ Peanut: 45.20%, Chickpea: 9.50%, Maize: 10.80%, Orange flesh sweet potato: 8.50%.

^3^ Peanut: 47.00%, Chickpea: 8.00%, Maize: 8.20%, Orange flesh sweet potato: 10.80%.

^4^ Peanut: 40.50%, Chickpea: 10.50%, Maize: 12.70%, Orange flesh sweet potato: 10.30%.

^5^ Peanut: 42.00%, Chickpea: 6.50%, Maize: 10.17%, Orange flesh sweet potato: 15.33%.

^6^ Peanut: 33.50%, Chickpea: 12.60%, Maize: 5.50%, Orange flesh sweet potato: 11.40%.

^7^ Peanut: 36.50%, Chickpea: 13.53%, Maize: 11.67%, Orange flesh sweet potato: 12.30%.

^8^ Peanut: 31.30%, Chickpea: 14.50%, Maize: 16.20%, Orange flesh sweet potato: 12.00%.

#### 3.4.2 Zinc Content

Zinc is an important mineral element during pregnancy for normal development (Oloniyo et al. 2021). The measured zinc content of the sample ranged from 5.01 to 6.74 mg per 100 g. PCMOFSP^8^ contained the highest zinc content (6.74 mg), while PCMOFSP^5^ had the lowest zinc content (5.01 mg). Significant differences were observed in the zinc content between the treatments (*p* < 0.05). Zinc is a vital mineral that plays a key role in preventing diarrhea in malnourished children (Veenemans et al. 2012). In this study, the zinc content of the food sample exceeded that of typical food blends used for managing MAM and was close to the recommended zinc levels (USAID Advancing Nutrition 2023).

#### 3.4.3 Iron Content

The measured iron content for the samples ranged from 8.39 to 11.34 mg/100 g. The iron content in PCMOFSP^5^ was significantly higher, whereas the lowest amount was found in ^1^PCMOFSP. There were statistical differences in the zinc contents among the treatments (*p* < 0.05). These values were higher than the recommended levels of iron for the management of MAM (9 mg/1000 kcal) (WHO 2016). This might be because the ingredients that we used for the development of PCMOFSP^5^ were rich in iron (Eloho et al. 2017). The iron content obtained in this study is higher than the comparable 1.06–1.48 g/100 g reported by Raru et al. (2022) for formulated composite flour from whole wheat, soy cake, rice bran, and oat bran. Iron is an essential mineral element for boosting immunity and preventing anemia (Borg et al. 2020).

#### 3.4.4 Potassium Content

The measured potassium content was significantly different among the treatments (*p* < 0.05). The potassium content of the sample ranged from 544.15 mg to 661.57 mg/100 g. The highest potassium content was found in PCMOFSP^8^ (661.57 mg). These levels were relatively similar to the recommended value (1400 mg/1000 kcal) (Chiopris et al. 2024). The therapeutic foods should contain an adequate amount of potassium to maintain renal and fecal excretion (FAO 2017). Potassium is a vital mineral element for the management of hypertension and helps with the absorption of iron in the body (Eloho et al. 2017).

#### 3.4.5 Phosphorous Content

The measured phosphorous value ranged from 442.54 to 451.84 mg/100 g. The least amount of phosphorus was found in PCMOFSP^1^, whereas the highest phosphorus content was found in PCMOFSP^4^. These values were similar to the values of phosphorus indicated in the study done by Gong et al. (2021). The values of these different minerals obtained in this study surpass those reported for soy plantain flour but are lower than the values shown for wheat, mushroom, and cassava composite flour (Ersino et al. 2018).

### 3.5 Sensory Acceptability

Sensory evaluation plays a crucial role in determining product quality, particularly regarding sensory acceptance and preference. In this study, therapeutic food samples were developed by blending the combination of peanut, chickpea, maize, and OFSP. These combinations were assessed to ensure optimal taste, texture, and overall appeal for the target consumer. The PCMOFSP^6^ samples is showed signifcance diffrence among the other samples (*p* < 0.05). The panelists were asked to rate each sample on specific sensory aspects, including color, aroma, taste, texture, and overall acceptability, to provide insights into consumer preference. The sensory acceptability evaluation of the food sample is shown in Table 6, with score values ranging from 3.28 to 4.31 for color, 3.30 to 4.73 for aroma, 3.27 to 4.19 for taste, 3.36 to 4.67 for texture, and 3.59 to 4.58 for overall acceptability. Significant differences were observed between the treatments (*p* < 0.05). The highest preferences for therapeutic food by color, aroma, taste, texture, and overall acceptability ranged from 4.31, 4.73, 4.19, 4.67, and 4.58, respectively. It was observed that the peanut‐based therapeutic food PCMOFSP^4^, prepared with the addition of 47% peanut flour, was liked most by sensory panelists as compared to the other combinations. The least preferred maize composite therapeutic food PCMOFSP^8^ ranged from 3.28, 3.30, 3.27, 3.36, and 3.59 for color, aroma, taste, texture, and overall acceptability, respectively. The sensory acceptability of the panelist preferences was increased as the ratio of peanuts increased in the composite flours. All products were rated greater than indicating that the color of the therapeutic foods was accepted or liked moderately by panelists. The proportions of ingredients such as peanuts, chickpeas, maize, orange‐fleshed sweet potatoes, sugar, sunflower oil, and pineapple powder result in variations in sensory qualities. Each of these ingredients contributes unique qualities in terms of flavor, texture, color, and overall appeal in the formulation.

**TABLE 6:** Sensory acceptability test.

Sample codes	Color	Aroma	Taste	Texture	Overall acceptability
PCMOFSP^1^	3.84 ± 0.46^ba^	3.30 ± 0.61^c^	3.69 ± 0.18^bc^	3.55 ± 0.66^c^	4.58 ± 0.14^a^
PCMOFSP^2^	3.94 ± 0.37^a^	4.03 ± 0.49^ba^	4.13 ± 0.28^ba^	4.26 ± 0.37^b^	4.30 ± 0.41^ba^
PCMOFSP^3^	4.07 ± 0.45^a^	4.18 ± 0.36^ba^	4.19 ± 0.39^ba^	4.56 ± 0.51^ba^	4.42 ± 0.45^ba^
PCMOFSP^4^	3.80 ± 0.68^ba^	4.03 ± 0.81^b^	3.79 ± 0.52^bc^	3.36 ± 0.55^c^	3.61 ± 0.32^c^
PCMOFSP^5^	4.11 ± 0.86^a^	4.02 ± 0.75^b^	3.78 ± 0.15^bc^	4.81 ± 0.22^a^	4.16 ± 0.49^ba^
PCMOFSP^6^	4.31 ± 0.40^a^	4.73 ± 0.35^a^	4.63 ± 0.43^a^	4.38 ± 0.33^ba^	4.09 ± 0.17^bac^
PCMOFSP^7^	3.79 ± 0.43^ba^	4.10 ± 0.67^ba^	3.27 ± 0.24^c^	4.67 ± 0.20^ba^	4.06 ± 0.03^bc^
PCMOFSP^8^	3.28 ± 0.29^b^	3.52 ± 0.39^bc^	3.41 ± 0.46^c^	3.49 ± 0.05^c^	3.59 ± 0.15^c^
LSD	**0.59**	**0.69**	**0.61**	**0.29**	**0.51**
CV	**8.67**	**10.00**	**8.95**	**6.54**	**7.07**

*Note:* Consumer perception (*n* = 50) of sensory attributes for formulated home‐based food. Mean different superscripts within the same column are significantly different (*p* < 0.05).

Abbreviations: CV, coefficient of variation; LSD, least significant difference.

^1^ Peanut: 44.00%, Chickpea: 11.00%, Maize: 7.00%, Orange flesh sweet potato: 12.00%.

^2^ Peanut: 45.20%, Chickpea: 9.50%, Maize: 10.80%, Orange flesh sweet potato: 8.50%.

^3^ Peanut: 47.00%, Chickpea: 8.00%, Maize: 8.20%, Orange flesh sweet potato: 10.80%.

^4^ Peanut: 40.50%, Chickpea: 10.50%, Maize: 12.70%, Orange flesh sweet potato: 10.30%.

^5^ Peanut: 42.00%, Chickpea: 6.50%, Maize: 10.17%, Orange flesh sweet potato: 15.33%.

^6^ Peanut: 33.50%, Chickpea: 12.60%, Maize: 5.50%, Orange flesh sweet potato: 11.40%.

^7^ Peanut: 36.50%, Chickpea: 13.53%, Maize: 11.67%, Orange flesh sweet potato: 12.30%.

^8^ Peanut: 31.30%, Chickpea: 14.50%, Maize: 16.20%, Orange flesh sweet potato: 12.00%.

### 3.6 Microbial Analysis

Table 7 presents the microbial load analysis of eight formulated home‐based foods. The sample PCMOFSP^6^, PCMOFSP^5^ and PCMOFSP^1^ are signifance difference among the samples (*p* < 0.05). The total plate count (TPC), an indicator of overall microbial load, ranged from 1011.87 CFU/g (PCMOFSP^4^) to 1097.08 CFU/g (PCMOFSP^8^), with PCMOFSP^8^ showing the highest load and PCMOFSP^4^ the lowest. Despite these differences, the low coefficient of variation (CV = 2.11%) highlights minimal variability among samples, reflecting consistent production or storage conditions. All total plate count values fall within the acceptable safety threshold for therapeutic foods, affirming the products' suitability for consumption. The total plate count (TPC) of the paste foods was recorded at 2.13 × 10^3^ CFU/g, well below the maximum limit of 10^4^ CFU/g set by the joint guidelines from WHO (Wojdat et al. 2005). The measured yeast and mold counts in the samples ranged from 33.67 CFU/g (PCMOFSP^4^) to 56.18 CFU/g (PCMOFSP^8^), with PCMOFSP^8^ exhibiting the highest count and PCMOFSP^4^ the lowest. The LSD value (3.68) indicates statistically significant differences between some samples. A coefficient of variation (CV = 4.81%) showed moderate variability, potentially attributed to differences in ingredients, handling, or storage practices. As yeasts and molds are naturally present in the environment, their presence in food can result from airborne contamination or inadequate equipment sanitation. The measured yeast and mold count of 42 CFU/g falls within the acceptable limit of 50 CFU/g, ensuring the product's microbial safety.

**TABLE 7:** Microbial analysis.

Sample codes	Total plate count (CFU/g)	Yeast and mold count
PCMOFSP^1^	1084.14 ± 0.59^a^	41.67 ± 3.06^d^
PCMOFSP^2^	1061.71 ± 0.59^bac^	41.41 ± 0.90^d^
PCMOFSP^3^	1045.17 ± 0.59^bdc^	36.47 ± 0.96^e^
PCMOFSP^4^	1011.87 ± 0.59^d^	33.67 ± 1.15^dc^
PCMOFSP^5^	1053.45 ± 0.59^bc^	43.83 ± 1.04^dc^
PCMOFSP^6^	1031.83 ± 0.59^dc^	45.78 ± 4.60^c^
PCMOFSP^7^	1083.55 ± 0.59b^a^	51.53 ± 1.27^b^
PCMOFSP^8^	1097.08 ± 0.59^a^	56.18 ± 0.12^a^
LSD	**3.03**	**3.68**
CV	**2.11**	**4.81**

*Note:* Mean different superscripts within the same column are significantly different (*p* < 0.05).

Abbreviations: CFU, colony‐forming units; CV, coefficient of variation; LSD, least significant difference.

^1^ Peanut: 44.00%, Chickpea: 11.00%, Maize: 7.00%, Orange flesh sweet potato: 12.00%.

^2^ Peanut: 45.20%, Chickpea: 9.50%, Maize: 10.80%, Orange flesh sweet potato: 8.50%.

^3^ Peanut: 47.00%, Chickpea: 8.00%, Maize: 8.20%, Orange flesh sweet potato: 10.80%.

^4^ Peanut: 40.50%, Chickpea: 10.50%, Maize: 12.70%, Orange flesh sweet potato: 10.30%.

^5^ Peanut: 42.00%, Chickpea: 6.50%, Maize: 10.17%, Orange flesh sweet potato: 15.33%.

^6^ Peanut: 33.50%, Chickpea: 12.60%, Maize: 5.50%, Orange flesh sweet potato: 11.40%.

^7^ Peanut: 36.50%, Chickpea: 13.53%, Maize: 11.67%, Orange flesh sweet potato: 12.30%.

^8^ Peanut: 31.30%, Chickpea: 14.50%, Maize: 16.20%, Orange flesh sweet potato: 12.00%.

## 4 Conclusions and Recommendations

The results show that home‐based therapeutic food for treating children aged 6–59 months with MAM was successfully developed using locally available ingredients such as peanut, chickpea, maize, and orange‐fleshed sweet potato. Except for calcium, the nutrients were within the recommended range of required nutrients. The PCMOFSP^6^, in particular, contains an excellent amount of nutrients for the treatment of MAM in children. As a result, PCMOFSP^6^ could be used in the rural community to manage children with MAM. The findings imply the need to enhance the production of food ingredients that have the potential to be used in home‐based therapeutic food to ensure availability and sustainability. A cost analysis of the superior home‐based therapeutic food should be conducted. Furthermore, fortifying the formulation with micronutrients like ascorbic acid and calcium is recommended. The findings also imply the need for enhancing the development of a guideline for home‐based therapeutic food for treating children with MAM from locally available food ingredients and enhancing the link between the agricultural and nutrition sectors. Integrate PCMOFSP^4^ into national MAM protocols, prioritizing rural communities with limited access to commercially ready‐to‐use therapeutic food.

## Author Contributions

**Gashaw Abebaw:** conceptualization (equal), data curation (equal), formal analysis (equal), funding acquisition (equal), investigation (equal), methodology (equal), resources (equal), software (equal), visualization (equal), writing – original draft (equal), writing – review and editing (equal). **Tefera Belachew:** conceptualization (equal), data curation (equal), resources (equal), software (equal), supervision (equal), visualization (equal), writing – review and editing (equal). **Welday Hailu:** conceptualization (equal), data curation (equal), resources (equal), software (equal), supervision (equal), visualization (equal), writing – review and editing (equal).

## Ethics Statement

This research received approval from the Institutional Review Board (IRB/314/2016) at Hawassa University College of Medicine and Health Sciences, as well as from the Institutional Research Ethics Review Board (IBR/23269/2024) at Arba Minch University's College of Medicine and Health Sciences.

## Conflicts of Interest

The authors declare no conflicts of interest.

## Data Availability

The authors declare that the data supporting the findings of this study are available within the paper. Should any raw data files be needed in another format, they are available from the corresponding author upon reasonable request.
